# Microbial Pathogenesis and Pathophysiology of Alzheimer's Disease: A Systematic Assessment of Microorganisms' Implications in the Neurodegenerative Disease

**DOI:** 10.3389/fnins.2021.648484

**Published:** 2021-04-28

**Authors:** Temitope Cyrus Ekundayo, Tosin Abiola Olasehinde, Kunle Okaiyeto, Anthony I. Okoh

**Affiliations:** ^1^SAMRC Microbial Water Quality Monitoring Centre, University of Fort Hare, Alice, South Africa; ^2^Applied and Environmental Microbiology Research Group, Department of Biochemistry and Microbiology, University of Fort Hare, Alice, South Africa; ^3^Department of Biological Sciences, University of Medical Sciences, Ondo, Nigeria; ^4^Nutrition and Toxicology Division, Food Technology Department, Federal Institute of Industrial Research Oshodi, Lagos, Nigeria; ^5^Department of Environmental Health Sciences, College of Medical and Health Sciences, University of Sharjah, Sharjah, United Arab Emirates

**Keywords:** microbial pathogenesis, pathophysiology, Alzheimer's disease, gut microbiota, neurodegenerative disease, yeast model

## Abstract

Microbial infections have been linked to the pathogenesis and pathophysiology of Alzheimer's disease (AD) and other neurodegenerative diseases. The present study aimed to synthesise and assess global evidence of microbial pathogenesis and pathophysiology in AD (MPP-AD) and associated neurodegenerative conditions using integrated science mapping and content analytics to explore the associated research landscape. Relevant MPP-AD documents were retrieved from Web of Science and Scopus according to PRISMA principles and analysed for productivity/trend linked to authors/countries, thematic conceptual framework, and international collaborative networks. A total of 258 documents published from 136 sources to 39.42 average citations/document were obtained on MPP-AD. The co-authors per document were 7.6, and the collaboration index was 5.71. The annual research outputs increased tremendously in the last 6 years from 2014 to 2019, accounting for 66% compared with records in the early years from 1982 to 1990 (16%). The USA (*n* = 71, freq. = 30.34%), United Kingdom (*n* = 32, freq. = 13.68%) and China (*n* = 27, 11.54%) ranked in first three positions in term of country's productivity. Four major international collaboration clusters were found in MPP-AD research. The country collaboration network in MPP-AD was characteristic of sparse interaction and acquaintanceship (density = 0.11, diameter = 4). Overall, international collaboration is globally inadequate [centralisation statistics: degree (40.5%), closeness (4%), betweenness (23%), and eigenvector (76.7%)] against the robust authors' collaboration index of 5.71 in MPP-AD research. Furthermore, four conceptual thematic frameworks (CTF) namely, CTF#1, roles of microbial/microbiome infection and dysbiosis in cognitive dysfunctions; CTF#2, bacterial infection specific roles in dementia; CTF#3, the use of yeast as a model system for studying MPP-AD and remediation therapy; and CFT#4, flow cytometry elucidation of amyloid-beta and aggregation in *Saccharomyces cerevisiae* model. Finally, aetiology-based mechanisms of MPP-AD, namely, gut microbiota, bacterial infection, and viral infection, were comprehensively discussed. This study provides an overview of MPP-AD and serves as a stepping stone for future preparedness in MPP-AD-related research.

## Introduction

Alzheimer's disease (AD) is a progressive neurological condition associated with degeneration of neurons, memory loss, learning impairment, and significant changes in character and behavioural activities (Deture and Dickson, 2019). AD is an age-related disease, although few cases have been identified in young people, the progression of the disease increases with age and has been reported to affect 10% of individuals between the age of 65 and 75 and about 32% of individuals above 80 years (Alzheimer's Association, 2016; Askarova et al., 2020). Currently, no cure has been identified to halt the progression of AD, which has been attributed to the complexity of its pathophysiology. Cholinergic dysfunction triggered by upregulation of acetylcholinesterase activity and depletion of the neurotransmitter acetylcholine has been identified as one of the causative factors of AD (Ferreira-Vieira et al., 2016). Furthermore, upregulation of beta-secretase activity, an important enzyme in the amyloidogenic pathway, triggers beta-amyloid peptide accumulation, which further aggregates into plaques (Götz et al., 2004; Vassar et al., 2009). These amyloid plaques may be released extracellularly or intracellularly and can trigger calcium imbalance, leakage of ions, and disruption of redox status, membrane potential, apoptosis, and synaptic loss (Reiss et al., 2018). It has also been hypothesised that AD may be associated with the accumulation of tau proteins and neurofibrillary tangles (Kolarova et al., 2012). Alterations in the phosphorylation of tau proteins reduce its capacity to stabilise tubulins, leading to the disorganisation of microtubules (Barbier et al., 2019). The aggregation of tau proteins also leads to the formation of neurofibrillary tangles (Brion, 1998).

Recently, the relationship between microbes (pathogens) and AD has been established (Pistollato et al., 2016). There are indications that bacterial and viral infections may trigger neurodegeneration associated with AD. A study carried out by Bu et al. (2015) revealed AD patients infected with some bacteria presented high serum levels of beta-amyloid peptide. Microbial infections caused by pathogens such as viruses (herpes simplex virus type-1 [HSV-1] and cytomegalovirus) (Renvoice and Hambling, 1984; Itzhaki et al., 1997) and bacteria (*Helicobacter pylori, Chlamydophila pneumonia*, and *Borrelia burgdorferi*) (Miklossy, 1993; Balin et al., 1998; Malaguarnera et al., 2004) have been linked with cognitive dysfunction. However, there exists no formal synthesis, assessment, or mapping of microbial pathogenesis and pathophysiology roles in AD. Therefore, this present study aimed to synthesise and assess global evidence and roles of microbial pathogens in AD using science mapping and content analytics. This study is the first study that applied integrated content analytics and systematic science mapping of microbial roles, pathogenesis, and pathophysiology in AD to explore the associated research landscape and forecast gaps for future research endeavours. Science mapping relied on mathematical and statistical techniques for quantitative and qualitative appraisal of previous studies in a domain to uncover research gaps for future research preparedness. The science mapping hinged on productivity/trend linked to authors and countries, thematic conceptual framework, thematic development, and international collaborative networks (intellectual-, resource-, skill-, personnel-sharing, etc.). This is expected to arouse interests, identify gaps, and create awareness for prospects in microbe-Alzheimer's disease research thrust.

## Methodology

### Acquisition of Data

**Figure 1:**
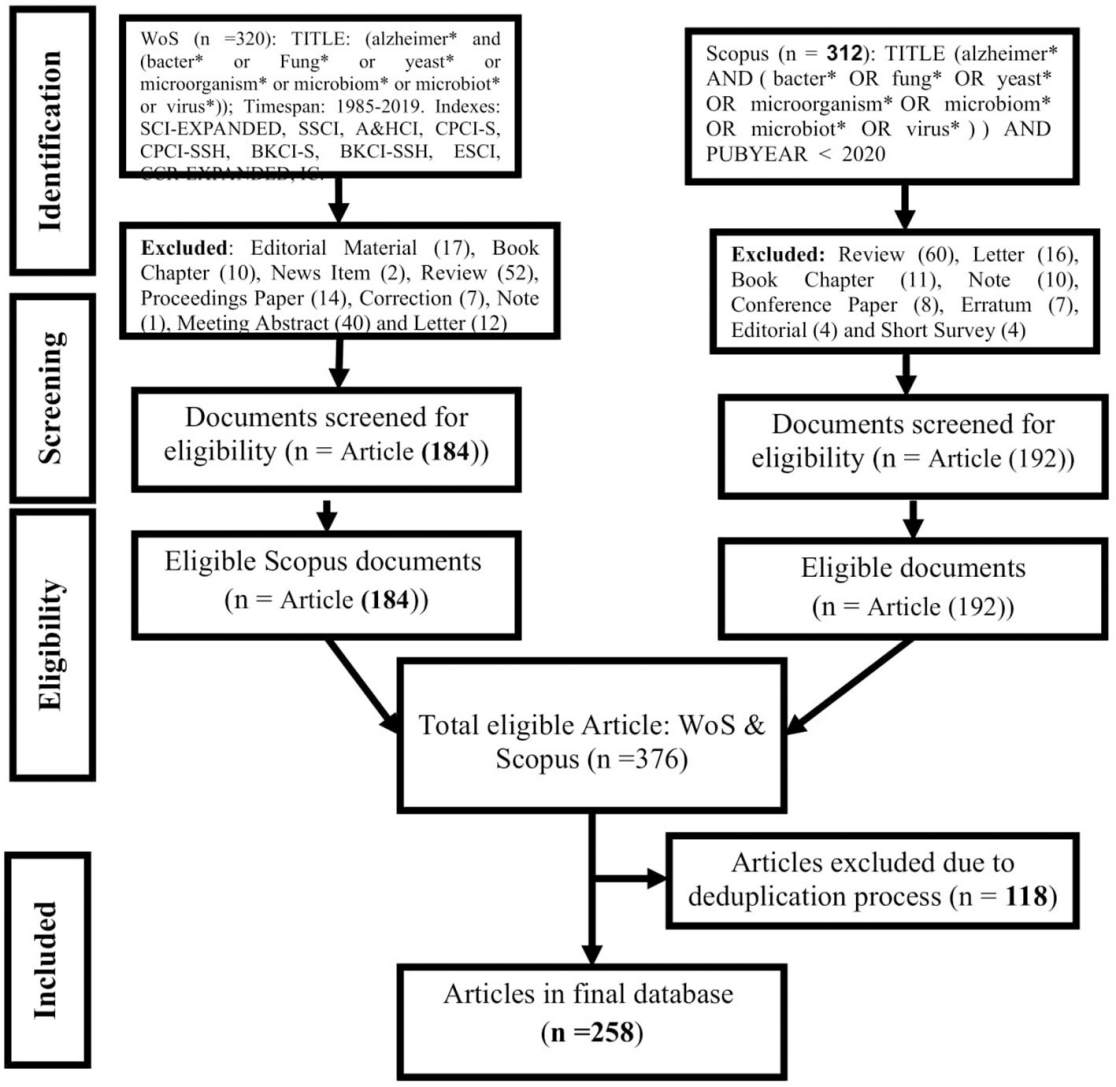
Procedural course for mining, screening, and selecting documents for qualitative and quantitative synthesis of MPP-AD related research.

For the systematic assessment of microbial pathogenesis and pathophysiology of Alzheimer diseases (MPP-AD), we searched the Web of Science (WoS) and Scopus for relevant studies using the guidelines of the “Preferred Reporting Items for Systematic Reviews and Meta-Analyses” (Moher et al., 2009). The search terms and the procedural details were as presented in Figure 1. The wildcard ^*^ was applied to improve the recovery of articles indexed with inflectional forms of the search terms in the databases. The returned articles were downloaded in the comma-separated (CSV Excel), BibTeX (bib), and Tab-delimited (Win, UTF-8) formats for pre-processing and further analyses.

### Data Analysis

The pooled articles from the databases were de-duplicated and associated variables normalised through ScientoPy package (Ruiz-Rosero et al., 2019) and bibliometrix package (Aria and Cuccurullo, 2017) using python and R environments, respectively. The normalisation protocol takes into consideration the harmonisation of spelling anomalies in author details [name and affiliations (institution and country)] and article details (keywords and source) to avoid inflationary or deflationary errors in the final outcomes of data analysis. The trend, descriptive, and productivity analyses of the standardised dataset were carried out in R programming environment. The descriptive variables consisted in the yearly production with the mean citations/articles and (co)-author indices (i.e., authors number, articles/author, number of appearances, single- and multi-authored articles, [co-] authors/article, and collaboration index). In contrast, productivity assessment was based on the first twenty top active individuals (authors) or entities (institutions, countries, and journals) and their corresponding H-index as well as citation rate.

Furthermore, the study identified the highest contributing articles related to MPP-AD. This was done through co-word analysis of author-keywords regulated to their root forms by Porter's stemming algorithm implemented in bibliometrix package coupled with an assessment of prevailing thematic areas using metric multidimensional scaling (MDS) based factor analysis (Aria and Cuccurullo, 2017; Igere and Ekundayo, 2020). Also, unsupervised k-means clustering of the MDS results was further performed for visualisation.

### Country Collaboration Mapping of MPP-AD-Related Research Landscape

Country network in MPP-AD-related research landscape was created using bibliometrix package (Aria and Cuccurullo, 2017) in the R environment as a square (adjacency) matrix of countries by publications. Representatively, the network's edges/nodes were participating nations, and the connecting curves/lines the bi-/multi-directional relationships/co-activities between them. The network was visually presented using Jaccard's similarity indexed Fruchterman force-directed layout (Jaccard, 1901). The main network statistics were also calculated for appropriate interpretations (Mao and Zhang, 2013).

## Results and Discussion

### Main Information

Table 1 depicts the results of global mapping of microbial pathogenesis and pathophysiology of Alzheimer's disease related-research over 38 years from 1982 to 2019 based on the information available on the WoS and Scopus. In the course of validation, out of the 376 articles extracted from the two databases, 116 documents were excluded due to duplication, and 258 documents were finally used for the analysis. These documents were published from 136 sources, comprising articles (*n* = 240), articles in book chapters (*n* = 9), and articles in proceedings papers (*n* = 9). A total of 1,391 prolific researchers authored these documents, with about 15 authors of single-authored documents and 1,376 authors of multi-authored documents, which means that multiple authors predominantly wrote the documents. The average citations per document were 39.42, and the documents consist of 1,692 keywords plus and 579 author's keywords. The documents per author was 0.185, and the authors per document were 5.39. The co-authors per document were 7.6, the collaboration index was 5.71, and this value indicated a high collaboration among the researchers that authored these documents.

**Table 1:** Main information on MPP-AD related research from 1982 to 2019.

**Variable**	**Rate/count**
Documents	258
Sources	136
Keywords Plus	1,692
Author's Keywords	579
Average citations/documents	39.42
Authors	1,391
Author Appearances	1,961
Authors of single-authored documents	15
Authors of multi-authored documents	1,376
Single-authored documents	17
Documents/Author	0.185
Authors/Document	5.39
Co-Authors/Documents	7.6
Collaboration Index	5.71
Document types	
Article	240
Article as book chapter	9
Article as proceedings paper	9

### Annual Research Outputs

**Figure 2:**
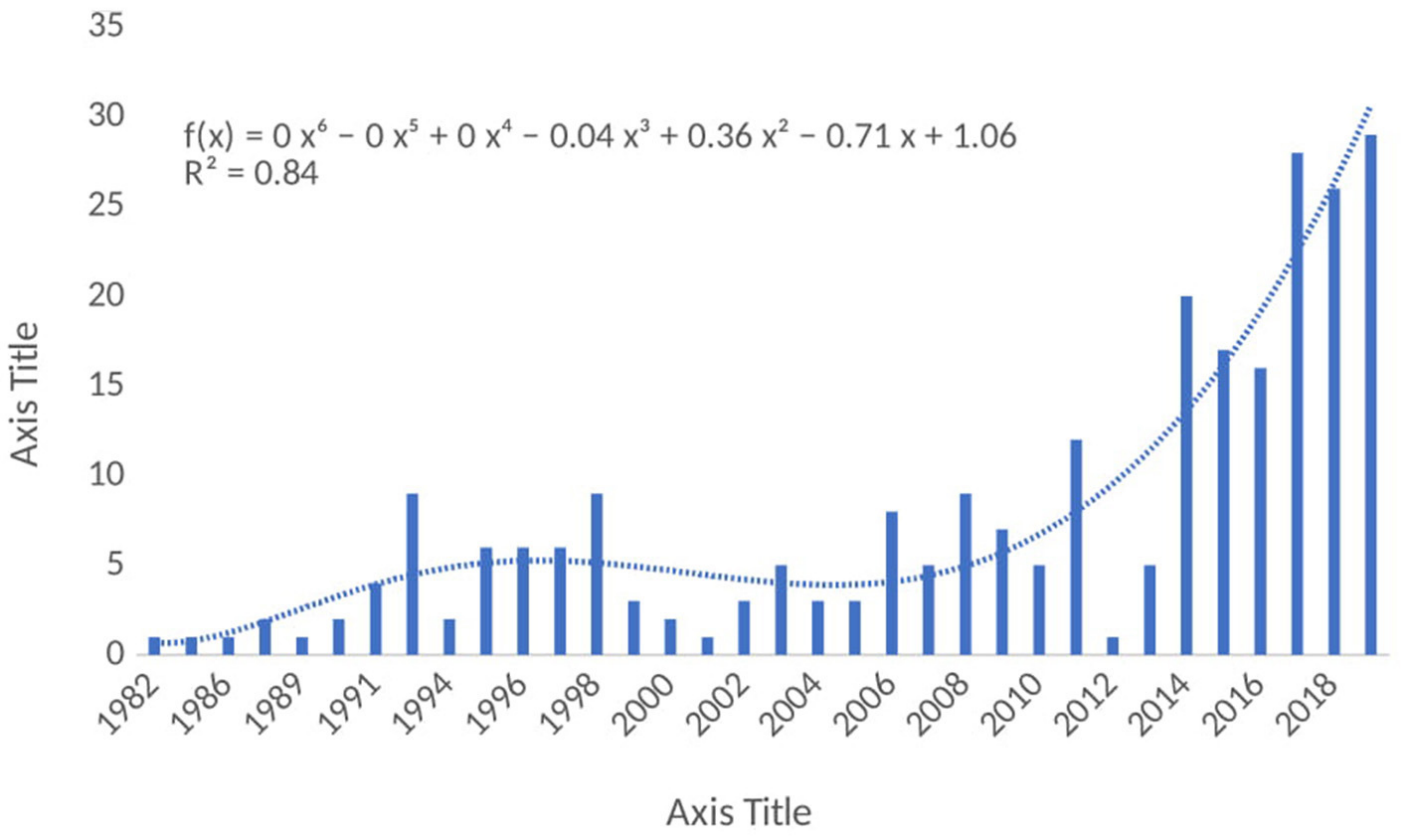
Annual research outputs on MPP-AD related research from 1982 to 2019.

The results of the annual research outputs in the MPP-AD research landscape were represented in Figure 2, an increase in the annual research outputs was observed from 1982 to 1991, although low outputs were observed in the early years and after that, there was a fluctuation in the outputs over the years. However, it could be observed that the annual research outputs increased tremendously in the last 6 years from 2014 to 2019 accounting for 66% as compared with the research output recorded in the early years from 1982 to 1990 (16%). As highlighted by the report of Grech and Rizk (2018), scientific research is characterised by relentless efforts coupled with incremental academic progress published in scholarly journals. Researchers always conduct research, and they acquire results from the research exercise. However, publishing the results of their findings has become crucial for the progress of academicians and researchers. Indeed, the growth in the struggle to publish has brought about bibliographical metrics that describe a journal's academic status as well as a scholar's productivity. However, several factors could be responsible for the fluctuation in the annual research outputs in this field. The progressive research trends noticed in the present study would help to make decision/policy on research directions on MPP-AD. Also, findings from these researches would help improve our understanding of some of the pathophysiological mechanisms of Alzheimer's disease.

### Most Productive Authors

The most prolific authors on MPP-AD-related research from 1982 to 2019 were evaluated, and the results are represented in Table 2. Among the listed authors, Itzhaki R was the most prolific authors with 21 articles with 1289 citations and h-index 15 with a publication start year from 1992 followed by Lukiw W (*n* = 12 articles and 563 citations, h-index 11) and publication start year from 2005. Macreadie I had 10 articles with 169 citations, h-index 6 from 2008, whereas Alonso R published nine articles with 329 citations with h-index 8 from 2014. Ball M started publishing in 1997 on the subject, and he had nine articles, 133 citations with h-index 5. According to Grech and Rizk (2018), “The h-index, or Hirsch index, measures the impact of an individual researcher.” The h-index is generally explored to determine the author's impact based on the number of published research articles and citations attributed to those publications. For example, a scholar with an h-index 10 implies that a scholar has published a minimum of 10 papers, and these papers have been received citations of no <10 times or equivalent. H-index can be used to compare the impact of research scholars. Although, h-index of researchers can be obtained in different databases, however, the calculation is somehow complicated, and it is usually difficult to use this metric to compare researchers from different fields because several limitations could influence an individual h-index in their respective field (Shanta et al., 2013; Grech and Rizk, 2018).

**Table 2:** Most productive authors on MPP-AD related research from 1982 to 2019.

**Rank**	**Author**	**Articles**	**h_index**	TC^*^	^*^PY_start
1	Itzhaki R	21	15	1,289	1992
2	Lukiw W	12	11	563	2005
3	Macreadie I	10	6	169	2008
4	Alonso R	9	8	329	2014
5	Ball M	9	5	133	1997
6	Carrasco L	9	8	329	2014
7	Pisa D	9	8	329	2014
8	Wilcock G	9	7	772	1992
9	Wozniak M	8	6	460	2004
10	Hill J	7	7	282	2005
11	Jamieson G	6	4	571	1992
12	Lin W	6	6	529	1995
13	Rabano A	6	6	260	2014
14	Varghese J	6	4	116	2008
15	Zhang H	6	4	233	2003
16	Zhao Y	6	4	224	2014
17	Bharadwaj P	5	4	99	2008
18	Kamal M	5	4	111	2014
19	Kotwal G	5	5	95	1998
20	Maitland N	5	3	144	2015
21	Shen L	5	3	203	1992
22	Tabira T	5	4	188	2017

* *TC, total citations; PY, publication year*.

Furthermore, this metric also affects the younger researchers with few publications in a particular field. They are not well-recognised compared to the established researchers with several publications that have received enormous citations. This means that the publication start year also influences the citations of a researcher. However, it is noteworthy that the researcher's h-index usually varies from one database to another, which could be due to the differences in the journal's indexes and the coverage period of the database.

### Most Cited Papers

Table 3 shows the most cited papers on MPP-AD. The paper authored by Crescenzi et al. (2002) published in Eur J Biochem received 426 citations with 23.67 citations per year, followed by a paper published in Lancet by Itzhaki et al. (1997) with 368 citations and 16 citations per year. Treusch et al. (2011) published the third most cited paper on the subject in Science, and the document has received 238 citations with 26.44 citations per year. Vogt et al. (2018) and Wozniak et al. (2009) published the most fourth and fifth most cited papers on Alzheimer's research with 204 citations, 68 citations per year, and 168 citations and 15.27 citations per year, respectively. Neuroscience research has been well-received in reputable and well-established journals with high impact factors. Interestingly, researchers always prefer to cite papers published in high-impact factor journals, and hence, papers published in these journals are cited more often (Lefaivre et al., 2011).

**Table 3:** Top most cited papers on MPP-AD related research from 1982 to 2019.

**Rank**	**Paper**	**TC**	**TCperYear**
1	Crescenzi et al., 2002, Eur J Biochem	426	23.67
2	Itzhaki et al., 1997, Lancet	368	16
3	Treusch et al., 2011, Science	238	26.44
4	Vogt et al., 2017, Sci Rep	204	68
5	Wozniak et al., 2009, J Pathol	168	15.27
6	Jamieson et al., 1991, J Med Virol	166	5.72
7	McLoughlin and Miller, 1996, Febs Lett	138	5.75
8	Pistollato et al., 2016, Nutr Rev	138	34.5
9	Kamer et al., 2009, J Neuroimmunol	119	10.82
10	Bhattacharjee and Lukiw, 2013, Front Cell Neurosci	118	16.857
11	Kamer et al., 2009, J Neuroimmunol	112	10.182
12	Zhan et al., 2016, Neurology	111	27.75
13	Hudry et al., 2010, Mol Ther	110	11
14	Wang et al., 2015, Mol Nutr Food Res	106	21.2
15	Letenneur et al., 2008, PLoS ONE	105	8.75
16	Jamieson et al., 1992, J Pathol	102	3.643
17	Lamb et al., 1997, Hum Mol Genet	100	4.348
18	Pisa et al., 2015, Sci Rep	99	19.8
19	Hill et al., 2014 Front Ageing Neurosci	96	16
20	Wozniak et al., 2005, J Med Virol	95	6.333
21	Jamieson et al., 1992, J Pathol	95	3.393

The citations received by these papers stemmed from their contributions to MPP-AD related research. For instance, Crescenzi et al. (2002) showed structural similarity and fusion between Alzheimer amyloid beta-peptide (1–42) and influenza hemagglutinin, suggesting a direct mechanism of neurotoxicity. Also, Itzhaki et al. (1997) found that simultaneous carriage of HSV1 (Herpes simplex virus type 1) in the brain and an apolipoprotein E epsilon 4 allele as a strong risk factor for AD. While Treusch et al. (2011) found a functional link between AD risk factors and A beta toxicity as well as endocytic trafficking in a yeast model, Vogt et al. (2018) revealed that alteration and differential abundance usually decreased Firmicutes/Bifidobacterium or increased Bacteroidetes correlated with cerebrospinal fluid biomarkers of AD.

### Author's Corresponding Countries

Table 4 depicts the detail of the corresponding author's on MPP-AD, and it could be seen that the developed countries dominated the network that are members of G20 countries. The USA was affiliated with 71 articles, and out of which, 55 were single country publications (SCP), and 16 were multiple countries publications (MCP). Similarly, United Kingdom had 32 articles (29 SCP and three MCP), China received 27 articles (26 SCP and one MCP), Italy had 14 articles (11 SCP and three MCP), and Australia had 13 articles (11 SCP and two MCP). It could be observed that the field is populated with researchers in the same country. This could be because these countries are developed nations with highly research leaders. Besides, the countries support research with sufficient government funding to purchase sophisticated equipment needed in their research. Interestingly, this provides intuitions about the significance of both financial support and skills in scientific research conceptualisation. The country's economic power directly impacts the budget allocated to the health system (Ye et al., 2018). However, we also observed MCP, although low as compared to the SCP. Nevertheless, it is worthy of documenting that research collaboration has greatly enhanced the research productivity recorded in this study. Another interesting thing to note is that China, a developing country, was listed among the developed countries, and this means that China holds a respectable position in MPP-AD related research. It could be observed that research on the subject has developed appreciably.

**Table 4:** Corresponding author's countries on MPP-AD related research from 1982 to 2019.

**S/n**	**Country**	**Articles**	**%Freq**	**SCP**	**MCP**	**MCP_Ratio (%)**
1	USA	71	30.34	55	16	22.54
2	United Kingdom	32	13.68	29	3	9.38
3	China	27	11.54	26	1	3.7
4	Italy	14	5.98	11	3	21.43
5	Australia	13	5.56	11	2	15.38
6	Spain	12	5.13	10	2	16.67
7	Japan	10	4.27	6	4	40
8	Mexico	6	2.56	6	0	0
9	Canada	5	2.14	4	1	20
10	Saudi Arabia	5	2.14	5	0	0
11	France	4	1.71	2	2	50
12	Germany	4	1.71	4	0	0
13	Sweden	4	1.71	3	1	25
14	Belgium	3	1.28	0	3	100
15	Korea	3	1.28	3	0	0
16	Russia	3	1.28	2	1	33.33
17	South Africa	3	1.28	0	3	100
18	Austria	2	0.86	1	1	50

Presently, there is no cure for Alzheimer's disease. However, several studies have been carried out by researchers in the field to elucidate the pathogenicity of the disease that could help in drug discovery and development. As a result, the phenomenal of the pathological mechanism of MPP-AD has become the subject of active research in the field. The pathological mechanisms that some researchers have elucidated include cholinergic neuron death and acetylcholine deficiency, accumulation of pathological misfolded tau, amyloid-β cascade hypothesis, oxidative stress and chronic inflammation, circadian dysrhythmia (Jaworski et al., 2011; Anand et al., 2012; Van Erum et al., 2018; Leuzy et al., 2019; Panza et al., 2019). Based on these few listed mechanisms, several drugs have been developed to inhibit or modulate the enzymes or proteins implicated in Alzheimer's disease. Several studies are ongoing to understand further the underlying mechanism of the disease that would assist in developing more effective therapeutic agents that may improve the treatment of Alzheimer's disease. The principal goals of Alzheimer's research are to determine when and how interventions can slow or stop cognitive decline in patients and identify early signs of decline and treatment response (Dong et al., 2019). However, more budget from these countries on the subject is highly imperative to allow more treatments to be carried out in clinical trials. Also, combination therapies that would allow multiple targets might be the future direction in this field.

Countries' citation analysis on MPP-AD was also carried, and the results are depicted in Table 5. The USA was the most cited country with 3,430 citations, average article citations of 48.31, followed by the United Kingdom with 2,044 citations, 63.88 average article citations. Italy, China, and Spain were the most third, fourth, and fifth most cited countries with 1,265, 591, and 549 citations, respectively (Table 5). This citation analysis was only based on the corresponding authors in the retrieved data from the two databases explored in this study.

**Table 5:** Total citations per country on MPP-AD research from 1982 to 2019.

**Country**	**Total citations**	**Average article citations**
USA	3,430	48.31
United Kingdom	2,044	63.88
Italy	1,265	90.36
China	591	21.89
Spain	549	45.75
Japan	284	28.4
Australia	275	21.15
Canada	243	48.6
France	235	58.75
Mexico	165	27.5
Korea	134	44.67
Saudi Arabia	111	22.2
Germany	72	18
Poland	72	72
Austria	46	23
South Africa	46	15.33
Sweden	36	9
Iran	28	28
Russia	21	7

### Most Relevant Sources on MPP-AD Research

The most relevant journals on MPP-AD are presented in Table 6. The top five journals were Journal of Alzheimer's Disease (*n* = 24 articles and 852 citations), PLoS ONE (eight articles, 393 citations), Handbook of Infection and Alzheimer's Disease (seven articles and 10 citations), Frontiers in Ageing Neuroscience (six articles and 292 citations) and Neurobiology of Ageing (six articles and 232 citations). The quality of a scientific journal is usually measured by its impact factor, and we observed that Alzheimer's research has received reputable attention in high impact factor journals. It is also remarkable to note that the first top journal was dedicated to Alzheimer's research. This is a multidisciplinary journal to facilitate understanding the aetiology, pathogenesis, and treatment of Alzheimer's disease. It has an impact factor of 3.909 and well-indexed in several scientific databases.

**Table 6:** Most relevant sources on MPP-AD related research from 1982 to 2019.

**Sources**	**Articles**	**h_index**	**TC**	**PY_start**
Journal of Alzheimers Disease	24	17	852	2004
PLoS ONE	8	6	393	2008
Handbook of Infection and Alzheimer's Disease	7	2	10	2017
Frontiers in Ageing Neuroscience	6	6	292	2014
Neurobiology of Ageing	6	6	232	1996
CNS and Neurological Disorders-Drug Targets	5	4	111	2014
Journal of Neuroimmunology	5	4	239	2009
Medical Hypotheses	5	4	83	2005
Faseb Journal	4	3	50	1996
Frontiers in Molecular Neuroscience	4	4	35	2017
Human Molecular Genetics	4	4	223	1995
Journal of Alzheimer's Disease	4	4	148	2004
Journal of Biological Chemistry	4	2	100	1998
Neurobiology of Disease	4	4	321	2003
Scientific Reports	4	3	374	2015
Vaccine	4	4	163	2006
Alzheimers and Dementia	3	3	98	2013
Archives of Gerontology and Geriatrics	3	3	16	1992
Frontiers in Microbiology	3	3	98	2016
Frontiers in Neurology	3	3	95	2014
Journal of Medical Virology	3	1	95	2005
Mechanisms of Ageing and Development	3	3	56	1992

### Countries' Collaboration

**Figure 3:**
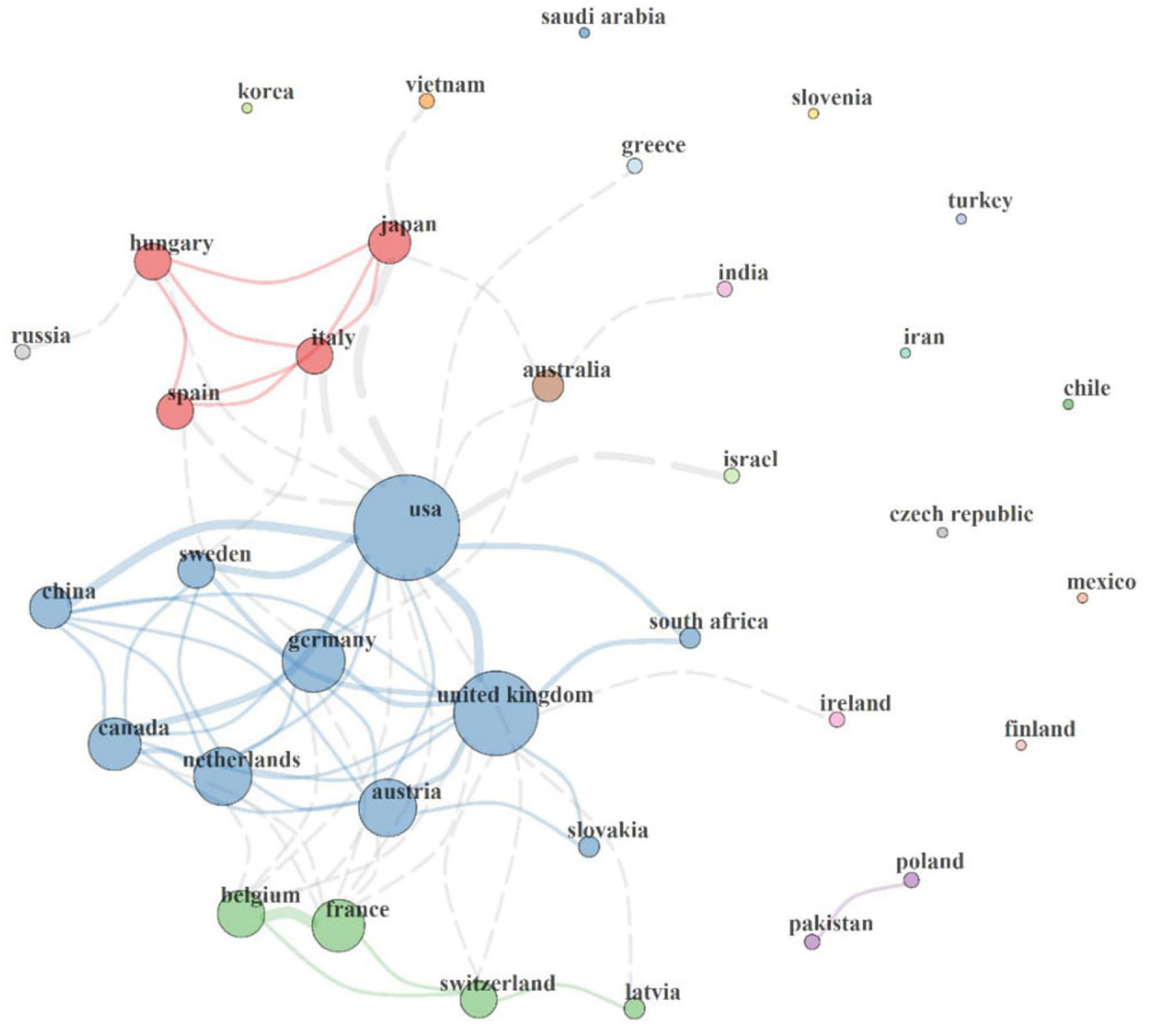
Country collaboration map in MPP-AD research. Network statistics: Size = 36, Density = 0.11, Transitivity = 0.535, Diameter = 4, Degree Centralisation = 0.405, Closeness Centralisation = 0.04, Betweenness Centralisation = 0.23, Eigenvector Centralisation = 0.767, Average path length = 2.053.

This section describes the collaboration network between the countries involved in MPP-AD research, and the results are represented in Figure 3. It could be observed that a circle represents each country, and of which the size of each circle indicates the total publications associated with each country. Furthermore, it is also worth noticing that each circle is connected to another by a line, which indicates the connexion between two countries. The thickness of each line connecting two countries shows the strength of the collaboration existing between them.

International collaboration clusters range between clusters of 2–10 countries. Four major collaboration clusters (2-nation purple, 4-nation green, 10-nation blue, and 4-nation red clusters) were found. The 2-nation purple cluster depicts collaboration between Pakistan and Poland. The 4-nation green cluster consisted of a collaboration involving Latvia, Belgium, Switzerland, and France, the 4-nation red cluster comprised scientific networks among Spain, Italy, Hungary, and Japan, and the 10-nation blue cluster agglomerate of Austria, China, Canada, Slovakia, South Africa, Sweden, Germany, USA, United Kingdom, and the Netherlands. Isolated countries on the map show that they lack international collaboration in the MPP-AD research landscape.

It could be served that the USA has the largest circle size with the highest collaboration with other countries. It has a direct link with the United Kingdom, Germany, Austria, Netherlands, Canada, China, Sweden, South Africa, Switzerland, Belgium, Spain, Hungary, Japan, Greece, Australia, and Israel. Similarly, the United Kingdom has a collaboration with the USA, Germany, Austria, Slovakia, Netherlands, Canada, China, France and Latvia. Some countries are not connected to the world leaders on MPP-AD research. Scientific evidence has highlighted that research collaboration results in an increase in research outputs, division of labour, exchange of ideas and skills, and research funding (Scarazzati and Wang, 2019).

Furthermore, based on the network statistics, 36 countries have a research focus on the MPP-AD research landscape (Size = 36). The country collaboration map's density and diameter were 0.11 and 4, characteristic of sparse global interaction, however, with acquaintanceship strength. Overall, the network transitivity and average path length was 53.5% and 2.053, respectively. The centralisation statistics, including degree (40.5%), closeness (4%), betweenness (23%), and eigenvector (76.7%), reveal a global inadequate international collaboration against the robust author collaboration index of 5.71 in the MPP-AD research landscape. For instance, eigenvector and centralisation reveal that 76.7% of nations exhibit independence or lack collaboration with other countries in the MPP-AD research landscape.

There is no doubt that global research on MPP-AD has increased enormously over a few decades. A progressive growth was witnessed with the substantial weight of scientific evidence in the volume of published articles on the subject study. Our findings collaborate with the report of Dong et al. (2019). It is quite interesting that most research originated in the United States, indicated by the country's significant role in MPP-AD research globally. Furthermore, with the rapid global growth in science and technology among the developed countries, findings from this present study put forward that healthcare expenditures are substantially related to economic power (Dong et al., 2019). Remarkably, these countries are well-positioned so that they have been recognised as main leaders with a significant impact in the field of neuroscience (Yeung et al., 2017). Although several drugs have been developed from developed countries that are currently used in the management of Alzheimer's patients, however, the adverse effect of toxicity associated with prolonged use of these drugs have shifted the attention of researchers to explore medicinal plants as potential sources for isolating new compounds to modulate some keys enzymes implicated in Alzheimer's disease (Howes et al., 2017; Olasehinde et al., 2017, 2019). Nonetheless, the toxic effect of herbal products due to the complex nature of herbal formulations, improper dosage, and unknown mechanisms of actions have delayed the breakthrough from medicinal plant research in the management of Alzheimer's disease.

**Figure 4:**
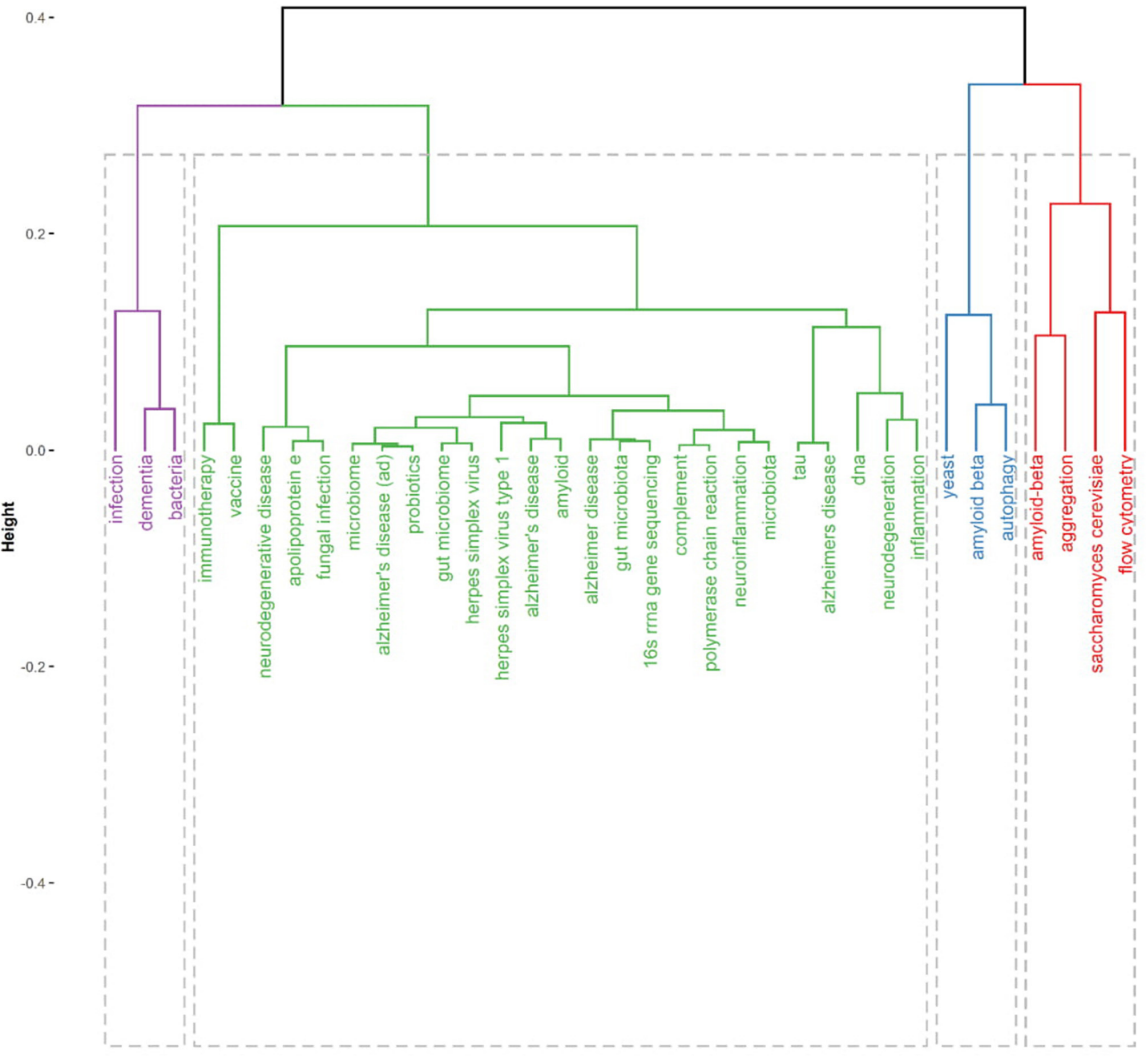
Thematic area and conceptual landscapes in MPP-AD research.

Figure 4 present thematic conceptual landscapes in MPP-AD research. Four conceptual thematic frameworks (CTF) were identified. The first CTF#1 (green cluster), named roles of microbial/microbiome infection and dysbiosis in cognitive dysfunctions. This CTF received the greatest attention. Disease pointer terms in CFT#1 consist of neurodegenerative disease, neurodegeneration, Alzheimer's disease, neuroinflammation, and inflammation. Disease biomarkers of a target in CTF#1 include amyloid-beta, apolipoprotein E, and tau protein. Aetiologic terms in CTF#1 include gut microbiota/microbiome, fungal infection, and herpes simplex virus. Also, the detection methods in the theme include complement/polymerase chain reaction and 16S rRNA sequencing. The therapeutic approaches in the CFT include vaccine, immunotherapy, and probiotics therapy. The second CTF#2 (purple cluster) is centred on bacteria and infection roles in dementia. It has been reported that bacterial (*Escherichia coli*) endotoxin potentiates beta-amyloid fibrillogenesis in AD and other forms of dementia (Asti, 2017). Also, bacterial lipopolysaccharide exhibits a complex array of pro-inflammatory neurotoxicity in AD (Zhao et al., 2017b). Further, an evident detection of spirochetal infection in the brain heightened the occurrence of AD by more than 10-fold; specifically, *Chlamydophila pneumoniae* infection was linked to greater than a 5-fold occurrence of AD (Maheshwari and Eslick, 2014). Thus, bacterial infection strongly and positively appears correlated with AD (Maheshwari and Eslick, 2017).

The third (CTF#3, blue cluster) is centred on the use/development of yeast as a model system for studying AD pathophysiology and remediation therapy based on amyloid-beta and autophagy. The fourth (CFT#4, red cluster) is centred on flow cytometry elucidation of amyloid-beta formation and aggregation in *Saccharomyces cerevisiae* model.

Yeast models (*Pichia pastoris* and *S. cerevisiae*) have been beneficial systems in understanding the pathogenesis and pathophysiology of AD and some neurodegenerative diseases. For instance, elucidation of *in vivo* amyloid beta-mediated aggregation has been demonstrated in yeast and tauopathy (Von Der Haar et al., 2007; Harris and Harris, 2015; Porzoor and Macreadie, 2016; Verduyckt et al., 2016). Specifically, humanised yeast AD models capable of expressing Aβ40 or Aβ42 under bioreactor cultivation have been reported (Chen et al., 2017). Aβ42 toxicity monitoring in AD has also been demonstrated in yeast models (De Vos et al., 2015; Fruhmann et al., 2018). It is worth noticing that the yeast-based vaccine model as a promising therapeutic approach for AD treatment, has been demonstrated. For example, yeast-based A-β epitope vaccine Y-5A15 successfully ameliorate memory and improve cognitive function in APP/PS1 transgenic mice by significantly rescuing synaptic deficits and reducing plaque burden, beta-amyloid levels, and glial activation (Liu et al., 2020).

## Aetiology-Based Mechanisms of MPP-AD

**Figure 5:**
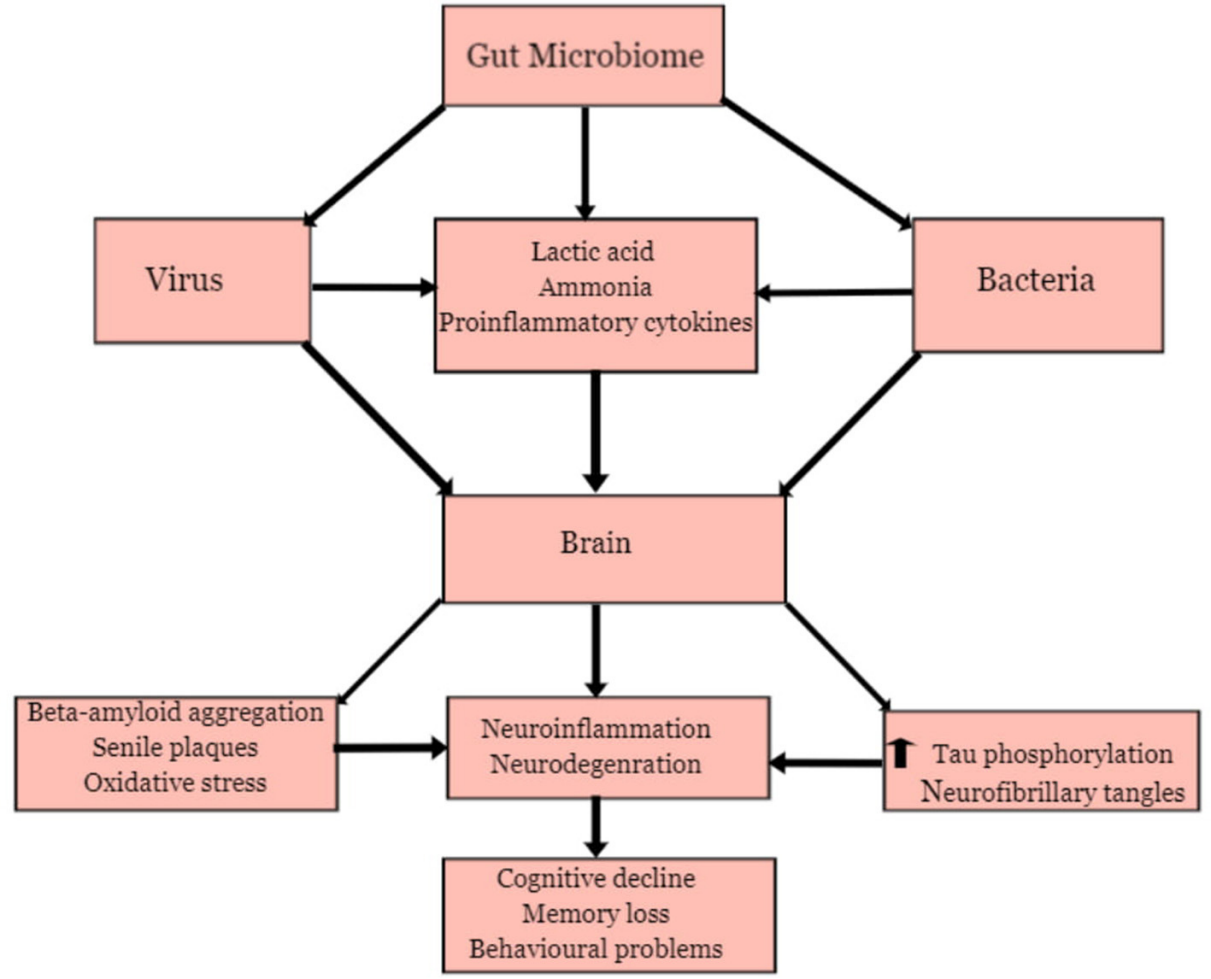
Microbial Pathogenesis and Pathophysiology of Alzheimer's disease.

Figure 5 present a general overview of MPP-AD. This shall be discussed comprehensively in the various groups of the aetiologic agents.

### Gut Microbiota and Alzheimer's Disease

Several microbes in the gut are responsible for regulating and maintaining the host's health (Angelucci et al., 2019). They participate in the physiology and development of host individuals to promote good health. At normal physiological conditions, the microbial community maintains a healthy and balanced ecosystem known as eubiosis and contributes to the regulation of the metabolism of neurotransmitters and synthesis of biological chemicals in the gut (Zhuang et al., 2018; Seo and Holtzman, 2020). However, disruption of the ecosystem, which could be triggered by high concentrations of antibiotics, suppression of the immune system, and alteration of the gastrointestinal barriers, may lead to pathological processes involving dysbiosis (Angelucci et al., 2019). Pro-inflammatory biomarkers produced during dysbiosis has been associated with the development of some neurological disorders (Bostanciklioglu, 2018; Shen et al., 2020). The community of microbes in the intestine such as bacteria and viruses has been identified as pathogens responsible for several acute and chronic diseases, affecting different organs from their primary site of infection *via* different mechanisms (Wozniak et al., 2007; Piacentini et al., 2011; Angelucci et al., 2019). The association between the gut microbiota and the brain has been proven due to the relationship between the intestine or enteric nervous system and the central nervous system (Zhuang et al., 2018; Angelucci et al., 2019). Furthermore, there is an exchange of biological substances through these two systems. These chemicals are transported *via* blood circulation and penetrate through the intestinal mucosa and blood-brain barriers. Hence, the gut microbiota can produce neurotoxic substances such as D-lactic acid and ammonia, which may induce neuronal damage (Galland, 2014). The inflammatory process may be triggered by releasing pro-inflammatory proteins such as cytokines and immune activators capable of inducing neuroinflammation (Bibi et al., 2014; Zhao et al., 2017a). The alteration in metabolic processes involved in the gut microbiota has been identified as a causative factor for anxiety, depression, cognitive impairment, learning, and behavioural problems that have been observed in some neurodegenerative diseases such as AD (Galland, 2014; Johnson and Foster, 2018; Angelucci et al., 2019).

### Bacterial Infections and AD

Recent reports have shown that gastrointestinal bacterial may play a contributory role in the development of AD (Bostanciklioglu, 2018; Li et al., 2019, 2020). The gut microbial community consists predominantly of different bacteria species, including Lactobacillus species and Bifidobacteria (Actinobacteria), Verrucomicrobia, Spirochetes Proteobacteria, Fusobacteria, Firmicutes, and Cyanobacteria (Bostanciklioglu, 2018). There are indications that bacterial infection may trigger some pathological processes that are associated with AD. Bacterial such as *Helicobacter pylori* (Wang et al., 2015), *Borrelia burgdorferi, Chlamydia pneumoniae* (Bu et al., 2015), *Escherichia coli, E. Shighella, E. rectale* (Cattaneo et al., 2017), *Bacteriodes fragilis* have been implicated in AD. Some of these bacteria species operate synergistically to influence infection burden on AD patients. One of the mechanisms by which these bacteria could trigger pathological involves the release of neurotoxic substances. Lactobacillus species and actinobacteria can metabolise glutamate to produce gamma-aminobutyric acid (GABA) in the central nervous system (Hudec et al., 2015; Bostanciklioglu, 2018). Moreover, high GABA levels in the intestine also influence an increase of GABA in the central nervous system. High levels of GABA in neuronal cells may trigger memory impairment, depression, and disruption in the formation of synapses (Mitew et al., 2013; Bostanciklioglu, 2018).

The gut bacteria also release inflammatory activators, beta-amyloid protein, and other neurotoxic substances that can disrupt the host immune system (Angelucci et al., 2019; Brown, 2019). These bacteria species can produce endotoxin, referred to as lipopolysaccharides, commonly found in the outer membrane of Gramme-negative bacteria (Brown, 2019). LPS-producing Gramme-negative bacteria are predominant in the gut, saliva, respiratory and urinary tracts, skin, dental plaque, and lungs (Zhan et al., 2018). During bacterial infection or alteration of gut microbiota metabolic processes due to inflammation, the concentration of LPS increases, which can induce neurodegeneration (Zhao et al., 2017a; Zhan et al., 2018). Previous studies revealed that administration of LPS in mice triggered microglial activation, memory problems, and neuronal damage (Hoogland et al., 2015). LPS also promotes beta-amyloid aggregation and the formation of senile plaques, which are important pathological processes involved in the development of AD (Brown, 2019). The study of Miklossy (2011) revealed the presence of senile-plaques with a resemblance to amyloid plaques after inhalation of *C. pneumoniae* in the mouse. Furthermore, exposure of spirochetes to neuronal and glial cells also showed amyloid-like pathology, which could be linked to neurodegeneration.

### Viral Infection and AD

Viruses, especially the Herpes simplex virus and cytomegalovirus, has been implicated in AD (Cheon et al., 2001; Wozniak et al., 2007). Viral infections may also trigger inflammatory markers that attack the neurons leading to neurodegeneration (De Chiara et al., 2012). An experimental investigation involving cells and animal models has shown that the Herpes simplex virus (HSV-1) may trigger the production of beta-amyloid peptide and elevation of tau phosphorylation which may lead to aggregation of beta-amyloid peptide, the formation of senile plaques, and neurofibrillary tangles (Lin et al., 2002; Wozniak et al., 2007). In rat's cortical neurons, HSV-1 triggered hyperexcitability and elevation of intracellular calcium signals, which induced alteration of amyloid precursor processing pathway and abnormal increase in amyloid-beta production (Piacentini et al., 2011). A study conducted by Wozniak et al. (2007) revealed that HSV triggered beta-amyloid peptide (Aβ40 and Aβ42) formation in human neuroblastoma cells. The human herpesvirus 6 (HHV-6) has been identified in the hippocampus and frontal and temporal cortex regions of AD patients' brains (Harris and Harris, 2015). Though there are indications that the HHV-6 virus is not directly associated with AD, reports have shown that this virus influences the damage caused by the HSV-1 virus and may induce lesions in the brain of infected patients (Lin et al., 2002).

## Study Limitations and Conclusion

The present study reveals a comprehensive science mapping of global research on MPP-AD. We observed an increase in annual productivity in MPP-AD across the study years. Itzhaki R and Lukiw W were the most prolific authors. The USA and the United Kingdom were the most relevant countries with the highest published articles and citations. We observed that the most powerful nations of the world are interested in MPP-AD and AD research. In addition, the Journal of Alzheimer's Disease and PLoS ONE were the most relevant journal on the subject. We also observed a high collaboration network between the researchers, although the research outputs were predominated by single country publication. Findings from this study would be valuable for younger researchers interested in joining the field in identifying potential collaborators. The strength of the study is shown in the first integrated content analysis and systematic science mapping of microbial roles in pathogenesis and pathophysiology of AD. The science mapping and the content analysis also offers a comprehensive overview of the MPP-AD research landscape.

However, the shortcoming of the science mapping consists of the use of WoS and Scopus, which might exclude documents in other databases that are not linked to the two databases. In addition, the focus of the analysis is on articles written in English and excluded those written in other languages. Furthermore, the analysis only measured the number of research articles published on the subject without considering the scientific quality of each article.

## Data Availability Statement

The original contributions presented in the study are included in the article/supplementary material, further inquiries can be directed to the corresponding author.

## Author Contributions

TE, TO, and KO designed the review and wrote the manuscript. TE searched the literature. All authors read and edited the manuscript.

## Conflict of Interest

The authors declare that the research was conducted in the absence of any commercial or financial relationships that could be construed as a potential conflict of interest.
